# Long-lasting olfactory dysfunction in COVID-19 patients

**DOI:** 10.1007/s00405-021-07153-1

**Published:** 2021-11-10

**Authors:** Bernhard Prem, David T. Liu, Gerold Besser, Gunjan Sharma, Laura E. Dultinger, Sissy V. Hofer, Martina M. Matiasczyk, Bertold Renner, Christian A. Mueller

**Affiliations:** 1 Department of Otorhinolaryngology, Head and Neck Surgery, Medical University of Vienna, Vienna General Hospital, 1090 Vienna, Austria; 2 Institute of Experimental and Clinical Pharmacology and Toxicology, Friedrich-Alexander Universität Erlangen-Nürnberg, Erlangen, Germany; 3 Institute of Clinical Pharmacology, Medical Faculty Carl Gustav Carus, Technische Universität Dresden, Dresden, Germany

**Keywords:** Anosmia, COVID-19, Hyposmia, Olfaction, SARS-CoV-2, Smell

## Abstract

**Objectives:**

Olfactory dysfunction (OD) is a common symptom of Coronavirus Disease 2019 (COVID-19). Although many patients have been reported to regain olfactory function within the first month, long-term observation reports vary. Therefore, we aimed to assess the course of chemosensory function in patients diagnosed with COVID-19 within 3–15 months after the infection.

**Methods:**

One hundred and two patients (71 females and 31 males; mean age 38.8 years) diagnosed with laboratory-confirmed COVID-19 and subjective OD participated in this single-center study 111–457 days after onset of OD. Patients first performed chemosensory tests at home, followed by psychophysical testing (Sniffin’ Sticks (TDI), 27-item Candy Smell Test (CST), Taste Strips Test (TST)) in the clinic. Questionnaires regarding importance of olfaction (IOQ) and olfactory-specific quality of life (QOD) were applied at both timepoints.

**Results:**

After a mean 216 days (SD 73; range 111–457) between OD onset and follow-up testing, the mean Sniffin’ Sticks (TDI) score was 27.1 points (SD 5.8; range 4.25–38.5): 4.0% were anosmic, 72.5% hyposmic, and 23.5% normosmic. At follow-up testing, 73.5% of patients reported improvement, 5.9% deterioration, and 20.6% no change in OD. Moreover, full recovery of self-perceived smell, flavor, and taste was not observed. According to questionnaires, the individual importance of smell did not change, but participants showed improvement in OD-related quality of life (*p* < 0.001) and had increased parosmia scores (*p* = 0.014) at follow-up.

**Conclusion:**

Our results show that long-lasting OD after SARS-CoV-2 infection is a common symptom. The majority of patients had OD in the range of hyposmia, which was confirmed by comprehensive smell tests.

## Introduction

Since the first reported human infection with severe acute respiratory syndrome coronavirus-2 (SARS-CoV-2) in December 2019 in Wuhan, China, [1] coronavirus disease 2019 (COVID-19) has spread throughout the world [2]. The course and symptoms of COVID-19 may differ considerably, ranging from mild almost asymptomatic disease to highly severe clinical progression, [3] with more than 3.1 million deceased by May 2021 [4]. Olfactory dysfunction (OD) and gustatory dysfunction are highly suspicious for COVID-19 and represent key symptoms in diagnosing infection with SARS-CoV-2 [5]. However, the prevalence of OD in COVID-19 patients differs due to the method of evaluation (anamnestic/subjective OD: 5% [6] and 58.8% [7] and psychophysical tests: 70% [8] and 98% [9]) or geographic region (54% in Europe, 51% in North America, 31% in Asia, and 10% in Australia) [10].

Chemosensory dysfunction has primarily been reported based on subjective patient reports through surveys and questionnaires [6,7]. Subsequently, loss of chemosensory function has been assessed by psychophysical smell/taste tests. These protocols range from self-made home-based methods to validated, comprehensive chemosensory tests (e.g., the orthonasal olfactory Sniffin’ Sticks test battery, threshold-discrimination–identification [TDI]) [8,9]. Reports on the duration of COVID-19-related OD have revealed that, 2 months after OD onset, 45% were diagnosed as hyposmic and 1% anosmic as measured by the TDI test [11]. Another study reported that, 5 weeks after symptom onset, 37% had persistent smell loss [12]. However, observations of OD related to COVID-19 over a more extended period are still rare.

As data regarding this topic are urgently needed for patient counselling, we aimed to prospectively assess COVID-19-related smell loss using validated psychophysical tests and validated patient-reported outcome measures (PROMs) several months after the onset of OD.

## Materials and methods

This study was approved by the ethics committee of the Medical University of Vienna (EK-No.: 1339/2020) and conducted according to the Declaration of Helsinki on biomedical research involving human subjects. All patients provided their written informed consent before participation.

### Patients

In the present monocentric study, a total of 102 patients with COVID-19-related OD (71f/31 m; mean age: 38.8 years; standard deviation (SD): 13.2 years) were recruited by notices placed at the campus of the Medical University of Vienna and several newspapers. SARS-CoV-2 infection was proven either by polymerase chain reaction (PCR) during acute sickness (*n* = 74) or at the follow-up appointment at the Department of Otorhinolaryngology at the Medical University of Vienna based on blood samples positive for antibodies (Roche^©^) against SARS-CoV-2 (*n* = 28). Patients < 18 or > 85 years of age or who had intolerance to sorbitol or fructose, head and neck tumours, dysphagia, head trauma, or neurological or sinonasal diseases were not eligible for the study. Furthermore, smell and taste loss before COVID-19 was considered as an exclusion criteria. According to the exclusion criteria, we had to rule out 19 interested subjects.

### Procedure

The study was divided into two parts. First, PROMs, self-rating of smell/flavor/taste function, a retronasal screening test (the 7-item Candy Smell Test [7-CST]), and a screening method to evaluate taste function (short taste strips test [STST]) were performed at home. All necessary papers and tests were distributed by post. The results and further explanation of part I have been published elsewhere [13]. Thirteen patients, who have participated in part I, did not take part for testing in the clinic due to lack of interest.

The present study’s investigations (part II) were carried out an average 216 days after the onset of OD. Two validated PROMs (the Questionnaire of Olfactory Disorders [QOD] and the Importance of Smell Questionnaire [IOQ]), self-assessment of smell/flavor/taste, the complete Sniffin’ Sticks test battery (TDI), the 27-item Candy Smell Test (CST), and the full Taste Strips Test (TST) were performed. Examinations were carried out between August 2020 and May 2021 at the Department of Otorhinolaryngology at the Medical University of Vienna.

Upon inclusion, all participants were instructed to perform smell training with different odourants on a daily basis [14]. Olfactory training consists of repeated exposure to four different odourants twice a day and is safe as well as low cost. Although its underlying mechanism is still unknown, the benefit has been proven in multiple studies [14].

### PROMs and questionnaires

The QOD assesses the olfactory-specific quality of life [15]. It consists of three parts evaluating negative statements (the extent to which patients suffer due to OD), positive statements (the degree to which patients cope with their OD), and parosmia score (the extent of qualitative OD symptomatology). Each answer is based on a 4-point Likert scale ranging from 0 (I agree) to 3 (I disagree). High results of negative statements (17 questions) are associated with a higher degree of suffering due to OD. Low results of positive statements (2 questions) suspect reduced capability of adjusting to OD. High scores on parosmia questions (4 questions) assume qualitative OD [16].

The IOQ consists of 20 questions related to the individual importance of smell [17,18]. The patients must pick one of four answers ranging from 0 (I totally disagree) to 3 (I totally agree). Higher individual significance of olfaction is associated with a higher score.

### Self-assessment of smell, flavor, and taste function

Participants classified their self-assessed chemosensory functions of smell (SAS), flavor (SAF), and taste (SAT) before the psychophysical tests. In addition, participants rated their chemosensory functions during home testing before infection (retrospectively) and on the day of home testing. Numeric rating scales ranged from 1 (very bad) to 10 (very good). All participants were informed about chemosensory functions to differentiate smell, flavor and taste.

### Chemosensory testing

Orthonasal olfactory function was measured by the comprehensive Sniffin’ Sticks test battery (TDI, Burghart Medical Technology, Wedel, Germany) [19]. This consists of three subtests: odor threshold (T), odor discrimination (D), and odor identification (I). Each subtest can achieve 16 points, resulting in a maximum score of 48. This test is based on a forced-choice paradigm and allows differentiation between normosmia, hyposmia, and functional anosmia. According to normative data, scores ≤ 16 represent anosmia, > 16 and < 31 represent hyposmia, and from 31 to 48 normosmia [20].The exact testing procedure is described elsewhere [19,21].

Retronasal olfactory function was assessed by the 27-item version [22] of the CST [23]. Each flavored candy consists of 500 mg sorbitol and a unique aroma. Candies are placed on the tongue, and the participant must pick one of four answers following the forced-choice paradigm. After each candy, the participant takes a sip of water or rinses their mouth with water. Studies have proven the general applicability of self-administration [22,24] and postal distribution [22].

By applying the TST, we sought to evaluate gustatory function [25]. Filter paper strips impregnated with four taste solutions (sweet, sour, salty, bitter) at four concentrations, as well as two blanks, formed the complete TST set. Patients had to choose one of five answers (sweet, sour, salty, bitter, or no taste). After each taste strip, the participants took a sip of water or rinsed their mouth with water. According to normative data, [25] scores between 9 and 16 represent normgeusia and scores between 1 and 8 hypogeusia. Thus, 0 points denotes ageusia. Detailed descriptions of the TST are given elsewhere [25].

### Statistical analysis

IBM SPSS 26.0 (IBM Corp., Armonk, NY, USA) and Graph-Prism 8.4.3 (GraphPad Software, Inc., La Jolla, CA, USA) were used for statistical analyses and graphical visualization. Utilizing histograms, we verified the normality of data distributions. Group comparisons were analyzed based on Student’s *t* test. For multiple group comparisons, we used one-way repeated-measures analysis of variance (rm-ANOVA), followed by Tukey’s post-hoc test. We utilized Pearson’s correlation coefficient (*r*) for bivariate correlations, where we interpreted *r* > 0.7 as strong, 0.4–0.7 as moderate, and < 0.4 as weak. *P* < 0.05 was considered significant.

## Results

Thirty-one men and 71 women with a mean age of 38.8 years (SD 13.2; range 18–68) performed home and follow-up testing of chemosensory function. The mean duration between the onset of OD and home testing was 57 days (SD 50). Between home testing and follow-up testing in the present investigation, another 159 days (SD 55) passed. Thus, 216 days (SD 74) elapsed from OD onset until follow-up testing (Table 1).

**Table 1:** Descriptive statistics (*N* = 102)

Descriptive statistics	
Gender	Female: 71/Male: 31
Age, years	Mean 38.8 (SD 13.2); range 18–68
Duration between onset of OD and home testing, days	Mean 57 (SD 50); range 7–374
Duration between home and follow-up testing, days	Mean 159 (SD 55); range 83–301
Duration between onset of OD and follow-up testing, days	Mean 216 (SD 74); range 111–457
Proof of COVID-19 infection via	PCR: 74 AB: 28

Self-Assessment	Before OD/Home testing/Follow-up [Mean (SD)]
Smell	9.3 (1.2)/4.1 (2.7)/6.0 (2.8)
Flavor	9.2 (1.2)/5.0 (2.8)/6.1 (2.9)
Taste	9.3 (1.4)/6.0 (2.6)/7.3 (2.2)

Questionnaires	Home testing/Follow-up [Mean (SD)]
IOQ	40.6 (7.7)/41.2 (7.4)
QOD-Parosmia	3.6 (2.4)/4.3 (3.4)
QOD-NS	16.3 (9.4)/12.1 (10.3)
QOD-PS	2.6 (1.5)/2.2 (1.6)

Psychophysical tests: home testing	[Mean (SD)]
7-CST	2.6 (2.1)
STST	3.5 (0.8)

*CST* 27-item Candy Smell Test; *7-CST* 7-item Candy Smell Test; *AB* Antibodies; *IOQ* Importance of Olfaction Questionnaire; *OD* olfactory dysfunction; *PCR* polymerase-chain-reaction; *QOD-NS* Questionnaire of Olfactory Disorders—Negative Statements; *QOD-Parosmia* Questionnaire of Olfactory Disorders—Parosmia Score; *QOD-PS* Questionnaire of Olfactory Disorders—Positive Statements; *SD* standard deviation; *STST* Suprathreshold Taste Strips Test; *TDI* threshold discrimination identification (Sniffin’ Sticks); *TST* Taste Strips Test

### Follow-up chemosensory testing

Orthonasal psychophysical testing using the TDI had a mean score of 27.1 points (SD 5.8; range 4.25–38.5). Compared to recently updated normative cutoff data, 23.5% were classified as normosmic, 72.5% hyposmic, and 4.0% anosmic (Table 2). Evaluation of retronasal olfactory function by the CST resulted in a mean score of 16.8 (SD 5.0; range 4–24). Assessment of taste function by the TST yielded a mean score of 11.0 (SD 2.6; range 3–15). According to normative cutoff data, normgeusia was found in 81.4% and hypogeusia in 18.6% of patients (Table 2).

**Table 2:** Results of follow-up psychophysical tests

	TDI	Distribution according to TDI*	CST	TST	Distribution according to TST^#^
All participants (*n* = 102)	27.1 (5.8)	Anosmic: 4.0% Hyposmic: 72.5% Normosmic: 23.5%	16.8 (5.0)	11.0 (2.6)	Hypogeusia: 18.6% Normgeusia: 81.4%
Group A (*n* = 32)	27.3^ns^ (6.6)	Anosmic: 6.3% Hyposmic: 65.6% Normosmic: 28.1%	17.6^ns^ (5.4)	11.2^ns^ (2.5)	Hypogeusia: 18.8% Normgeusia: 81.3%
Group B (*n* = 70)	26.7^ns^ (6.4)	Anosmic: 4.3% Hyposmic: 74.3% Normosmic: 21.4%	16.4^ns^ (4.9)	10.8^ns^ (2.7)	Hypogeusia: 18.6% Normgeusia: 81.4%

Data are given as mean (SD) unless otherwise noted

Group A: implemented home testing within 30 days after the onset of olfactory dysfunction (OD); Group B: performed home testing beyond 30 days after the onset of OD

*CST* 27-item Candy Smell Test; *SD* standard deviation; *TDI* threshold discrimination identification (Sniffin’ Sticks); *TST* Taste Strips Test

*According to Oleskiewicz et al. [20]

^#^According to Mueller et al. [25]

^ns^No significant difference between Group A and Group B comparing each psychophysical smell/taste test with each other based on Students *t* test

Furthermore, no correlation was found between the chemosensory test results (TDI, CST, and TST) and the duration between onset of OD and follow-up testing (*p* = 0.116, *p* = 0.390, *p* = 0.543, respectively).

To evaluate differences in the dependence of OD duration, we separated the study cohort into two groups. Group A consisted of participants who implemented home testing within 30 days after the onset of OD. Therefore, patients who completed home testing ≥ 31 days after the start of OD formed Group B. Comparing the results of the two groups did not reveal significant differences regarding the chemosensory tests (TDI: *p* = 0.827; CST: *p* = 0.282; TST: *p* = 0.430) (Table 2).

### Correlations between self-reported and psychophysically evaluated chemosensory functions

Individual self-assessment of smell and TDI results at follow-up revealed a moderate correlation (*r*_102_ = 0.429; *p* < 0.001). Furthermore, weak but significant correspondence between SAT and TST was found (*r*_102_ = 0.258; *p* = 0.009). Moreover, analyzing SAF and CST showed a moderate correlation (*r*_102_ = 0.551; *p* < 0.001).

### Course of self-perceived chemosensory function

**Fig. 1:**
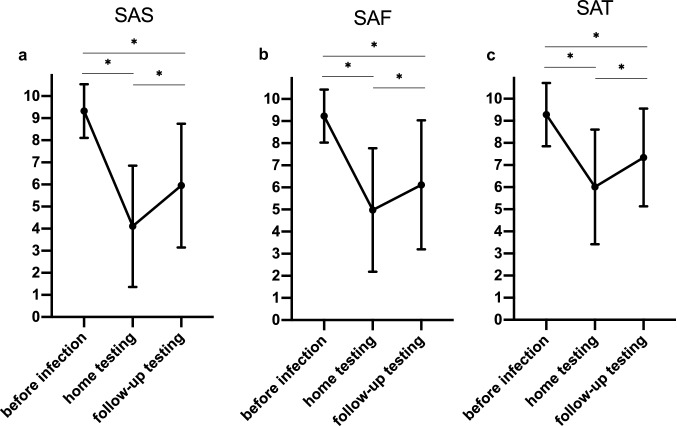
Course of self-reported chemosensory functions. Scale: 1 (very bad) to 10 (very good). *significant difference based on one-way repeated-measures ANOVA, followed by Tukey’s post-hoc test. *SAS* self-assessment of smell; *SAF* self-assessment of flavor; *SAT* self-assessment of taste

We also compared the self-assessment of chemosensory function over the course of time (Fig. 1). One-way rm-ANOVA for SAS, SAF, and SAT showed significant differences across the observational period (*F* (101, 202) = 2.243, *p* < 0.001; *F* (101, 202) = 2.138, *p* < 0.001; and *F* (101, 202) = 1.845, *p* < 0.001, respectively). Tukey’s post-hoc test revealed significant deterioration of all qualities (smell, flavor, and taste) within the acute phase of SARS-CoV-2 infection. Furthermore, Tukey’s post-hoc test showed significant improvement in all chemosensory qualities when evaluated during follow-up testing. Nevertheless, analyzing the self-assessment of these qualities before OD and during the follow-up appointment, Tukey’s post-hoc test still revealed a significant difference.

### Course of the IOQ

To evaluate the importance of olfaction over time, we applied the IOQ during home and follow-up testing (Table 1). The comparison (paired *t* test) of the individual IOQ scores revealed no significant difference.

### Course of daily life problems due to OD

We applied the QOD to evaluate the daily life problems caused by OD and whether patients adapt over time (Table 1). The positive statement score (the capability of adjusting to OD) and the score for negative statements (the extent to which patients suffer from OD) decreased significantly (*p* = 0.029; *p* < 0.001, respectively). However, the parosmia score increased significantly between home and follow-up testing (*p* = 0.014).

## Discussion

Investigating olfactory dysfunction in COVID-19 patients over a long period of time, we had three main findings. First, measuring olfactory function revealed hyposmia in the majority of investigated patients up to 15 months after symptom onset. Second, self-assessed smell function improved significantly compared to initial loss, but did not reach the level of function before the disease. Third, according to questionnaires, the importance of smell did not change with time, but parosmia increased and the extent of suffering due to OD decreased significantly.

Few studies have assessed olfactory function in patients with SARS-CoV-2-related smell loss using PROMs and psychophysical tests [12,26–28]. Solely asking for smell function frequently leads to incorrect results [29–31]. Therefore, it is important to combine PROMs and tests of chemosensory function to uncover the course of the regeneration of smell loss [14].

Among our patients, only 23.5% achieved normosmia after a mean duration of smell loss of 216 days, with the vast majority (76.5%) still exhibiting some degree of OD. Our study provides further evidence that a considerable group of patients with SARS-CoV-2-related smell loss does not recover rapidly, which is in contrast to the pandemic’s initial reports [12, 26, 28]. Most reports early in the pandemic suggested that most patients regain normal smell function after a short period of time. Reiter et al. reported that 72% recovered from OD within 1 month based on anamnestic evaluation [32]. Other colleagues reported similar results: 63% within 1–4 weeks, [26] 62% within 5 weeks, [12] and 75% within 2 months [28]. Few long-term observations exist: Niklaasen et al. reported that most of the affected people recovered within 28 days, but approximately 27% still suffered from different degrees of OD by applying TDI 28–169 days after the onset of OD [27]. Another multicenter study reported that approximately 95% regained their ability to smell after 6 months by implementing psychophysical smell tests [33]. By applying the culturally adapted University of Pennsylvania Smell Identification Test, Boscolo-Rizzo et al. found that 60% still suffered from different degrees of OD 6 months after the onset of OD [34]. A 6-month online follow-up survey showed that approximately 36% of patients affected by COVID-19-associated OD complained about persistent reduced olfactory function [35].

Concerning the degree of persistent OD, we found that most patients scored within the range of hyposmia. Only 4% of the investigated patients were diagnosed with functional anosmia 3–15 months after disease onset. This leads to the assumption that OD due to infection with SARS-CoV-2, though lasting a long time in some patients, may not permanently disrupt the olfactory epithelium in most cases and leave a subgroup of olfactory neurons intact. This could be because specific ACE receptors responsible for the invasion of SARS-CoV-2 are primarily located on olfactory sustentacular cells, [36,37] leaving olfactory receptor neurons less affected.

Although we cannot report the exact percentage of patients with persistent smell loss (longer than 4 weeks), it seems obvious that a notable proportion of infected patients do not recover as quickly as initially reported. Potential support for this theory is that we did not find differences between patients included within the first 30 days of OD and those included later. Consequently, patients with rapid recovery of smell function who experienced OD only for a brief period of time (maximum 2 weeks) may not have participated in the present study. The results of the self-assessment of smell, taste, and flavor in our patients may support these considerations, because the majority of self-perceived functions failed to recover fully. During acute infection and home testing, the self-ratings of chemosensory functions decreased significantly. However, at follow-up testing, they increased significantly but still differed significantly from the pre-OD values. However, in this case our study has one limitation: patients rated their chemosensory functions before infection retrospectively, at the same time when they evaluated the functions of smell, flavor, and taste during home testing. Therefore, it may be possible that patients rated their chemosensory abilities retrospectively too well. Furthermore, consistent with previous studies, our results revealed only weak to moderate correlations between self-assessed and psychophysical evaluations of chemosensory functions [38].

Moreover, recruitment of these studies’ patients’ needs to be discussed as potential selection bias. In our study, patients with proven SARS-CoV-2 infection and subjective OD contacted us for inclusion. Thus, mainly olfactory highly attentive and interested patients, who suffer severely from smell malfunction may be included into this study. Furthermore, the gender distribution may allow conclusion that more women than men are interested into individual olfactory function and improvement of its dysfunction.

Regarding the olfactory-related quality of life, the negative statement score of the QOD decreased significantly, meaning less suffering from OD. Furthermore, the parosmia score increased significantly, indicating the emergence of qualitative OD due to SARS-CoV-2 infection over time. The individual importance of olfaction did not change within 3–15 months. This may indicate that, even after several months of decreased olfactory function, patients desired recovery of their chemosensory function and consequently seemed highly interested in adequate treatment.

According to the current Position Paper on Olfactory Dysfunction [14], olfactory training is the recommended therapy for post-infectious OD. Based on multiple studies that reveal beneficial features of olfactory training [39–42], daily practicing with four odourants has become standard recommendation of COVID-19 related OD. Despite smell training, different centers studied the effect of cortisone—intranasal as well as oral—on the outcome regarding OD. In a case–control study with 50 patients in each group (olfactory training solely vs. olfactory training and intranasal cortisone) the self-assessment of smell did not differ between the groups [41]. Furthermore, Kasiri et al. revealed no significant difference concerning SAS and psychophysical tests (the Iran Smell Identification Test) between intervention (intranasal cortisone and olfactory training) and control (solely olfactory training) group [42]. Whereas, another case–control study showed a significant improvement of psychophysically evaluated smell function after olfactory training and 10 days of oral cortisone. The results of the control group (only smell training) did not change significantly [40]. Nevertheless, besides the proven effectiveness of olfactory training, the value of cortisone needs to be evaluated in larger studies.

As the pandemic is still ongoing in most affected countries and vaccination of the majority of the population will last months, to even years, knowledge and recognition of the course of the disease is of great importance regarding patient management. Therefore, as long as therapy for post-infectious smell loss is restricted to smell training, patients need to be counseled appropriately. Currently, based on our results, SARS-CoV-2-related smell loss seems to last longer in select patients than previously thought, with the majority of patients in the hyposmic range. Anosmia, the complete loss of the sense of smell, seems to be rare. Consequently, smell training specifically should be advocated, as the degree of OD constitutes a prognostic factor regarding the success of olfactory training [43]. Nevertheless, further research is urgently needed regarding factors that influence prolonged COVID-19-related smell loss.

## Conclusion

Olfactory dysfunction is a common and well-known symptom of COVID-19. Although previous investigations have shown that most patients recover within the first few weeks, our results highlight that knowledge about long-lasting OD related to SARS-CoV-2 infection is an essential aspect of patient management.
